# Effect of Smoke Exposure on Gene Expression in Bone Healing around Implants Coated with Nanohydroxyapatite

**DOI:** 10.3390/nano12213737

**Published:** 2022-10-25

**Authors:** Felipe Nunes, Paula Oliveira, Edmara Bergamo, Per Kjellin, Arthur Novaes, Bruna Ghiraldini, Fabio Bezerra, Sergio Scombatti de Souza

**Affiliations:** 1 Department of Oral and Maxillofacial Surgery and Periodontology, School of Dentistry of Ribeirão Preto, University of São Paulo, Ribeirão Preto 14021-630, SP, Brazil; 2 Department of Periodontology, School of Dentistry, University Center of State of Para, Belem 66060-575, PA, Brazil; 3 Department of Prosthodontics and Periodontology, School of Dentistry of Bauru, University of São Paulo, Bauru 17012-901, SP, Brazil; 4 Promimic AB, AstraZeneca BioventureHub, 431 83 Mölndal, Sweden

**Keywords:** gene expression, dental implant, implant surface, nanohydroxyapatite, animal study, smoking

## Abstract

This study evaluated the effect of smoke exposure on the expression of genes related to bone metabolism in implants coated with nanohydroxyapatite (NHA). A total of 36 rats were exposed to cigarette smoke for 60 days. The animals were allocated into three groups: machined implants (MAC), dual acid-etched implants (DAE), and NHA-coated implants (NHA). Implants were installed in the left tibia of the rats after 30 days of smoke exposure. The implants were retrieved 7 and 30 days after implantation, and the adjacent bone analyzed using a real-time polymerase chain reaction for gene expression of alkaline phosphatase (ALP), osteopontin (OPN), receptor activator of the nuclear factor kappa ligand (RANKL), osteoprotegerin (OPG), the RANKL/OPG ratio, osteocalcin (OCN) and runt-related transcription factor 2 (Runx2). After 7 days, Runx2, OPN and OPG expression demonstrated significantly higher levels for the NHA surface treatment relative to DAE and MAC surfaces. NHA presented the lowest RANKL and RANKL/OPG levels. After 30 days, NHA-coated implants showed significantly higher levels of Runx2, ALP, OPN, OPG, OC, RANKL and RANKL/OPG relative to DAE and MAC implants. The results indicated a greater osteogenic and high osteoclastic activity around NHA implants, in comparison to DAE and MAC implants.

## 1. Introduction

Oral rehabilitation using osseointegrated implants has been considered a predictable treatment modality for partially and totally edentulous patients [1], with more than 90% survival rates after 10 years of follow-up [2]. Nonetheless, osseointegration establishment and maintenance is highly dependent on the clinical procedure and a healthy bone metabolism, which has been shown to be impaired by several factors, including three-dimensional implant position in the alveolar ridge, inter-implant and inter-dental distance [3], occlusal loading [4], local and systemic conditions [5], as well as deleterious habits, such as smoking [6].

According to the world cancer report of the World Health Organization (WHO), more than 1 billion people, approximately 20% of the worlds adult population in 2014, were smokers [7]. The total number of smokers is progressively increasing, and the individuals are smoking more cigarettes. This pandemic smoking habit has become one of the main public health threats, since previous studies have reported several negative effects on the entire body, including compromised wound healing [8]. In fact, some of the chemicals present in the cigarette smoke have a potential deleterious effect, such as acrolein and acetaldehyde, which inhibit the proliferation and adhesion of gingival fibroblasts [9], carbon monoxide, which leads to an impaired tissue oxygenation [10], and hydrogen cyanide, which inhibits oxidative metabolism and oxygen transport at the cellular level [11].

Among the more than 4000 substances found in cigarette smoke, nicotine has been considered the most critical for the risk of adverse health-related effects [12]. Nicotine has been shown to decrease the blood supply by releasing catecholamines, resulting in vasoconstriction and, consequently, decreased tissue perfusion [13,14]. Nicotine also reduces the proliferation of hemoglobin, macrophages, and fibroblasts, which are important elements for tissue healing [10]. It also decreases the osteogenic activity, thereby increasing the periodontal and peri-implant bone loss, potentially increasing the risk of implant failure [15]. Previous studies have demonstrated increased implant failure rates in smokers (11.28%) relative to non-smokers (4.76%) [16], as well as a higher prevalence of biologic complications, such as peri-implantitis and marginal bone loss [17,18].

Profound changes in dental implants principles, engineering design, and clinical practice have opened up new prospects for the treatment of such a susceptible population. Modifications in the implant microgeometry, along with implant surface topography and chemistry, have been proposed to hasten bone healing around implants, especially for challenging clinical situations [19], such as low-quality bone and the treatment of compromised health-related patients. Alterations in the surface texture and chemistry can be achieved by using a large set of methods, including blasting, acid etching, and chemical and electrochemical coating [20]. Complex implant surfaces have been shown to increase the available surface area for protein adsorption, resulting in more sites for cell fixation, and to modulate host response by maximizing osseoconduction through increased migration and the adhesion of osteogenic cells to the implant surface [21,22,23]. Recent developments have shifted the interests to nanoscale textured surfaces and the resultant impact on modulating osteogenic capability [24,25]. Nanostructured implant surfaces have been of great importance during the initial stages of bone healing, where increased biomolecular level interactions improve protein adsorption, osseoconduction, and osseoinduction phenomena [26]. Moreover, nanostructured calcium phosphate (CaP) coatings, including the nanohydroxyapatite, on implant surfaces have been demonstrated to accelerate osseointegration and improve bone mechanical properties relative to conventional surface modifications through the positive influence of the intrinsic chemistry osteoconductive properties of CaP, mimicking the composition and structure of the surrounding bone [27].

There are several proteins that are essential in the bone re-modeling process following implant placement, where the runt-related transcription factor 2 (Runx2), alkaline phosphatase (ALP), osteocalcin (OC), osteopontin (OPN), osteoprotegerin (OPG), receptor activator of nuclear factor Kappa-B (RANK), and receptor activator of nuclear factor Kappa ligand (RANK-L) are of particular importance. Runx2 is the main transcription factor of early osteoblast differentiation and bone formation [28,29]. ALP is a glycoprotein produced by osteoblasts that plays an important role in the initial phase of differentiation, being necessary for osteoid formation and matrix mineralization [30,31,32]. OC is a non-collagenous extracellular matrix protein, secreted by osteoblasts, with a high affinity for calcium, essential for skeletal morphogenesis and bone formation [33]. OPN is also a non-collagenous extracellular matrix protein expressed by different cell types, including osteoblasts and osteoclasts, which interacts with cell surface integrins to regulate cell adhesion, migration and proliferation, controlling bone formation and calcification [34,35,36]. The OPG/RANKL/RANK system plays a central role in paracrine regulation and osteoclast function, where OPG binds to RANKL, both secreted by osteoblasts, preventing RANKL binding to RANK in the osteoclast membrane and inhibiting bone resorption [37,38].

Nanotexturization has been shown to positively regulate the expression of osteogenic genes, accelerating and increasing bone formation when compared to microstructured and machined implants [39,40,41,42]. Similarly, nanohydroxyapatite-coated implants have been demonstrated to significantly improved the expression of osteogenic genes, as well as high osteoclastic activity, indicating an increased potential for bone remodeling around this type of surface [43]. Considering the potential benefits of a nanoscale hydroxyapatite-coated surface on hastening osseointegration, understanding its benefits in the presence of long-term cigarette smoke exposure becomes paramount. Therefore, this study aimed to evaluate the relative gene expression levels of bone metabolism mediators around nanohydroxyapatite-coated implants placed in the tibia of rats exposed to cigarette smoke, in comparison with dual acid-etched and machined surfaces. The postulated null hypothesis was that implant surface treatment would not influence gene expression levels in the presence of smoke exposure.

## 2. Materials and Methods

This study was conducted under the ethical approval of the Animal Experimentation Ethics Committee of the University of Sao Paulo-Ribeirao Preto School of Dentistry (protocol number: 2015.1.910.58.5).

### 2.1. Animals and Experimental Groups

Adult male Wistar rats (Rattus norvegicus albinus), weighing between 200 g and 250 g, were selected in the study. The animals were maintained in appropriate plastic cages with food and water ad libitum, in a 12 h cycle of light and darkness, and a temperature of 23 ± 1 °C during the experimental period. Prior to surgical procedures, the animals remained in the facility for 7 days to acclimate.

The animals were randomly divided into 3 experimental groups by a computer program, separated by two subgroups of timepoints, according to the implant surfaces treatment, as follows: (i) machined implants with no surface treatment (MAC), (ii) dual acid-etched implants (DAE, nitric acid and sulfuric acid), and (iii) nanohydroxyapatite-coated implants (NHA). The DAE surface was obtained from a machined implant surface that received baths of nitric acid followed by sulfuric acid, in a micro corrosion process. For the NHA surface treatment, a DAE implant surface was processed: coating liquid containing a dispersion of nanohydroxyapatite crystals was applied on top of the implant to be coated, and the implant was placed on a spin coater device. The implant was rotated at 2600 rpm for 3 s, for homogenization of the liquid over the entire surface, and allowed to dry for 10 min at room temperature. The implant was then placed in an oven at 450 °C for 5 min, to remove the surfactants and to sinter and improve the adhesion of the HA crystals [44,45]. The treatment resulted in an evenly dispersed layer on the implant surface, 20–40 nm thick, of hydroxyapatite nanosized crystals.

The information of each animal allocation according to implant type and in vivo period was kept in sealed envelopes and revealed immediately before surgical procedures to ensure that the operator was blinded to the studied factors. Considering that implant surface treatment and time were the independent variables evaluated, the minimum sample size calculated based on preliminary data of the current study (20% of the sample size) to obtain a statistical test power of 80% and a 5% alpha error within an effect size of 0.7 was 36 in total, 12 per experimental group (6 for each timepoint) (G*Power 3.1, HHU University, Düsseldorf, Germany).

### 2.2. Smoking Induction

**Figure 1:**
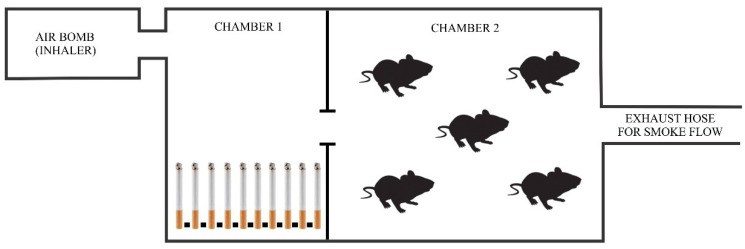
Schematic drawing representing the smoke exposure mechanism. The cigarettes were placed in chamber 1 and the rats remained in chamber 2 during the period of exposure to cigarette smoke.

The animal cigarette device consisted of transparent acrylic boxes with dimensions of 45 × 20 × 20 cm, composed of two chambers connected by an orifice. In the first chamber, lit cigarettes were placed, and the air was pumped by means of a small compressor (adapted from an air inhaler) to the second chamber, where the rats were maintained. In the second chamber there was also an orifice that allowed the flow of the pumped air to the external environment [46,47] (Figure 1). Initially, the animals went through an adaptation period through cigarette smoke exposure for 1 min three times in the first day, 7 min three times in the second day, and 8 min three times daily from the third day until the sacrifice, a methodology adapted from the Nociti Junior et al., in 2002 [48].

### 2.3. Surgical Procedures

After 30 days of smoke exposure three times daily, implant placement was planned. After anesthesia, 1 mL of blood was collected from each rat, extracted by cardiac venipuncture, for plasma analysis of cotinine levels, which is a nicotine metabolite that has been used as a reliable marker to investigate the effects of smoking and smoking cessation in in vivo research. Cotinine levels were also analyzed 7 and 30 days after implant placement, prior to animal euthanasia. Quantification of cotinine was performed with the use of an enzyme-linked immunosorbent assay (ELISA) kit (mouse/rat cotinine ELISA, OriGene Technologies Inc., Rockville, MD, USA), following manufacturer’s instructions.

**Figure 2:**
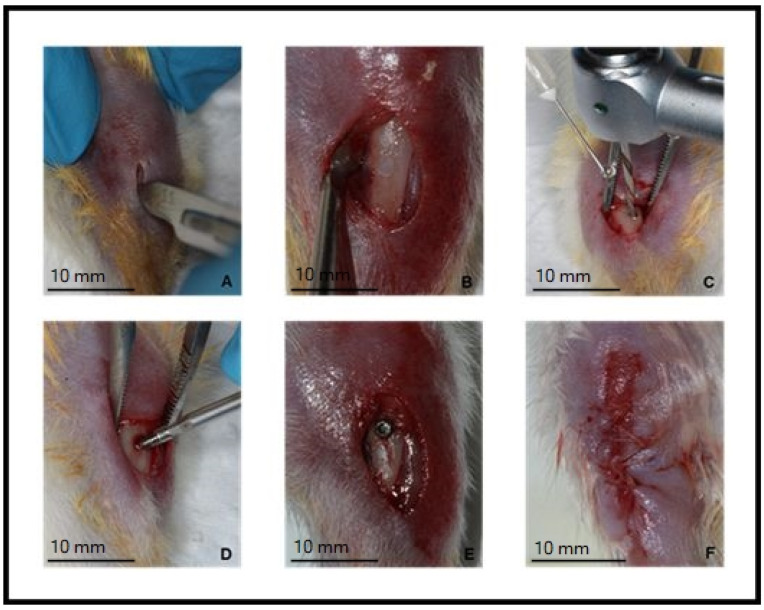
Surgical sequence for implant installation. (**A**)—Incision of approximately 1.5 cm parallel to the tibial axis; (**B**)—flap reflected; (**C**)—drilling under constant saline irrigation; (**D**)—installation of the implant; (**E**)—implant positioned; (**F**)—sutured flap.

Implant placement was conducted based on the protocol used by Prado et al., 2006 [49]. General anesthesia was obtained by the combination of 50 mg/1 kg of ketamine hydrochloride (Agener União Ltd., Sao Paulo, SP, Brazil) and 10 mg/1 kg of xylazine hydrochloride (Rompum; Bayer SA, Sao Paulo, SP, Brazil) through intramuscular injection. After anesthesia, trichotomy and antisepsis with a 1% povidone-iodine solution (Rioquímica Ind. Farmacêutica, São Jose do Rio Preto, Brazil) were performed in the left tibia. Subsequently, a 1.5 cm incision was performed using a type 15 scalpel blade (Swann-Morton, Sheffield, England, UK) parallel to the long axis of the tibia (Figure 2A). Tissues were dissected until the periosteum was exposed, which was then detached to expose the tibia bone (Figure 2B). The osteotomy for the implant placement was performed using a 1.0 mm diameter and 15 mm length pilot drill (S.I.N-Implant System, Sao Paulo, SP, Brazil) under constant irrigation with saline solution, according to the manufacturer recommendations (Figure 2C). The implants were installed using a 0.9 mm diameter wrench (Figure 2D), until the threads were completely inserted into the cortical bone (Figure 2E). Then, wounds were closed with 5-0-coated absorbable suture (Vicryl Ethicon 5.0, Johnson Prod., São José dos Campos, São Paulo, Brasil) (Figure 2F). After surgery, a single dose of anti-inflammatory (ketoprofen 0.05 mg/kg), and antibiotic (penicillin 24,000 IU/kg) were administered. The animals were maintained in the appropriate plastic cages with water and food ad libitum, and observed daily for symptoms of pain, dehiscence, infection, limited movement, lack of appetite or weight loss.

The animals were euthanized with a 150 mg/kg dosage of 2.5% sodium thiopentate, intraperitoneally (Thiopentax, Cristália, Itapira, SP, Brazil) 7 and 30 days after implant placement (6 of each of the experimental group per timepoint). The implants were retrieved using a 3 mm diameter trephine, stored in liquid nitrogen and then maintained in the −80-degree freezer for gene expression analysis.

### 2.4. Gene Expression Analysis

The samples consisted of bone fragments of two tibiae, which were macerated, with the same implant surface treatment and time in vivo, totaling the need for 18 specimens. The total RNA was extracted with Trizol reagent (Life Technologies, Invitrogen, Carlsbad, CA, USA) followed by SV Total RNA Isolation System (Promega, Madison, WV, USA) following the manufacturer’s instructions. The total RNA concentration and purity were evaluated by spectrophotometry in a NanoVue device (GE Healthcare, Uppsala, Sweden). The reading was performed at wavelengths of 260 nm, 280 nm, and 230 nm, to obtain the concentration of RNA/µL and contamination by proteins and phenol, respectively. Only samples that presented the ratio 260:280 and 260:230 greater than 1.8 were considered for the quantitative real-time polymerase chain reaction assays (qRT-PCR). The integrity of the total RNA was analyzed using the 2100 Bioanalyzer equipment (Agilent Technologies, Stockport, Manchester, UK). Samples with RNA Integrity Number (RIN) values equal or higher than 7 were used to synthesize complementary DNA (cDNA) from 1 μg of total RNA, using cDNA High-Capacity cDNA Reverse Transcription Kit (Applied Biosystems, Foster City, CA, USA), following the manufacturer’s instructions. RT-PCR was conducted in a StepOnePlus RT-PCR system (Life Technologies, Carlsbad, CA, USA), and for each reaction 5 μL of Taqman^®^ Gene Expression Master Mix (2X) 2, 0.5 μL of (20X) Taqman^®^ Gene Expression Master Mix, and 4.5 μL of cDNA (11.25 ng) for a final volume of 10 µL/reaction were used. The thermal cycling specifications consisted of 2 min at 50 °C, 10 min at 95 °C, forty cycles of 15 s at 95 °C, and 1 min at 60 °C (denaturation and extension).

The results were analyzed based on the value of Ct (cycle threshold), the point corresponding to the number of cycles in which the amplification of the samples reaches a threshold that is determined between the level of fluorescence of the negative controls and the exponential amplification phase of the samples, allowing the quantitative analysis of the expression of the evaluated factor. The relative gene expression was measured in reference to constitutive expression gene glyceraldehyde-3-phosphate dehydrogenase (GAPDH), used as a positive control of the amplification reaction. The expression levels of the constitutive gene were used to normalize the expression levels of the target gene and a negative sample (water), exposed to the reaction with each Taqman probe used. The quantification of gene expression data was performed using the 2 DDCT method [50]. The gene expression evaluation analyzed alkaline phosphatase (ALP), osteopontin (OPN), receptor activator of the nuclear factor kappa ligand (RANKL), osteoprotegerin (OPG), the RANKL/OPG ratio, osteocalcin (OCN) and runt-related transcription factor 2 (Runx2). All gene expression analyses were performed by a single examiner, blinded to the experimental groups.

### 2.5. Statistical Analysis

Plasma cotinine and gene expression data analyses showed a normal distribution (Shapiro–Wilk test, *p* > 0.05) and homogeneity of variance across groups (Levene test, *p* > 0.25). All data were collected, aligned along a general linear model with fixed factors of time (7 and 30 days) and surface treatment (MAC, DAE and NAH). After administering a significant omnibus test, post hoc comparison of the experimental groups’ means was accomplished using Tukey’s test. Data are presented as a function of mean values and 95% confidence interval (mean ± 95% CI). The analyses were performed using SPSS (IBM SPSS 23, IBM Corp., Armonk, NY, USA).

## 3. Results

No adverse events occurred during or following surgeries, and a clinically healthy appearance was observed in the surgical areas during healing for all animals.

### 3.1. Cotinine Analysis

**Figure 3:**
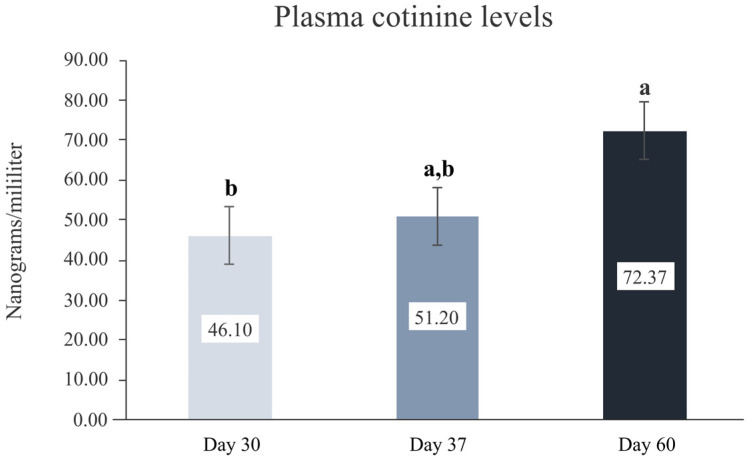
Plasma cotinine level of rats, in nanograms per milliliter, at different times after exposure to cigarette smoke. Mean values, error bars show 95% confidence interval. Different letters indicate statistically significant difference between groups.

Plasma analysis of cotinine levels demonstrated the cumulative effect of cigarette smoke exposure on the animals. Cotinine levels were 46.10 ± 7.26 ng/mL of blood plasma after 30 days of smoke induction, 51.20 ± 7.26 ng/mL after 37 days and 72.37 ± 7.26 ng/mL after 60 days, with a statistically significant difference between the mean value of day 60 when compared to day 30 (*p* < 0.001) (Figure 3).

### 3.2. Gene Expression Analysis

#### 3.2.1. Runt-Related Transcription Factor 2 (Runx2)

**Figure 4:**
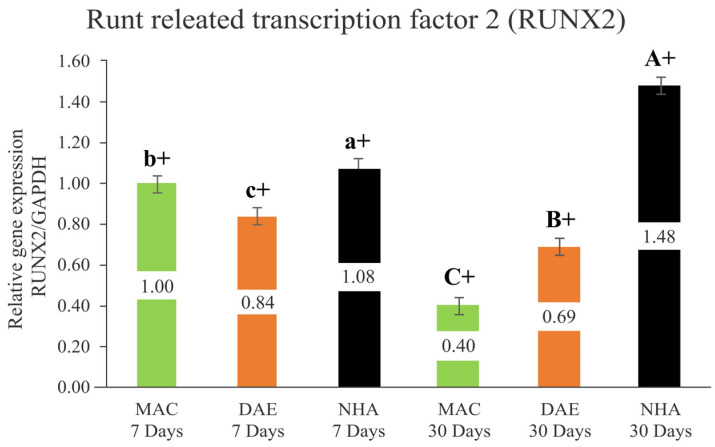
Relative expression of Runx2/GAPDH (Runx2/GAPDH) in all groups (mean values, error bars show 95% confidence interval). Lower case letters represent the analysis of 7 days, and capital letters the analysis of 30 days. Different letters indicate a statistically significant difference between groups. The + sign indicates a statistically significant difference (MAC: *p* < 0.001; DAE: *p* < 0.001; NHA: *p* < 0.001) for intragroup analyses, considering the same surface treatment and different assessment times.

After 7 days, Runx2 expression demonstrated higher levels for the NHA surface treatment (NHA 7D = 1.08 ± 0.04) relative to DAE (DAE 7D = 0.85 ± 0.04) and MAC surfaces (MAC 7D = 1.00 ± 0.04), all statistically significant different (*p* < 0.005). Similarly, after 30 days of healing, implants that presented NHA surface coating showed the highest Runx2 expression levels (NHA 30D = 1.48 ± 0.04), followed by DAE (DAE 30D = 0.70 ± 0.04) and MAC implants (MAC 30D = 0.40 ± 0.04), all statistically significant different (*p* < 0.001) While Runx2 expression levels significantly increased from 7 to 30 days of healing for NAH implants (*p* < 0.001), its expression levels significantly decrease in a temporal perspective for MAC and DAE implants (*p* < 0.001) (Figure 4).

#### 3.2.2. Alkaline Phosphatase (ALP)

**Figure 5:**
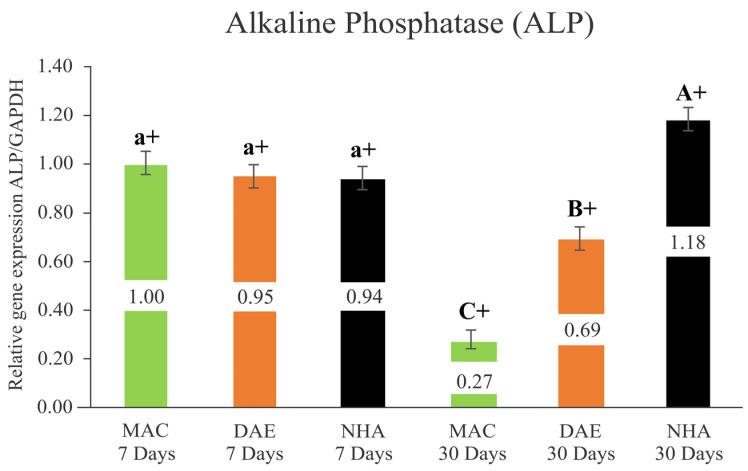
Relative expression of alkaline phosphatase/GAPDH (ALP/GAPDH) in all groups (mean +95% confidence interval). Lower case letters represent the analysis of 7 days, and capital letters the analysis of 30 days. Different letters indicate a statistically significant difference between groups. The + sign indicates a statistically significant difference (MAC: *p* < 0.001; DAE: *p* < 0.001; NHA: *p* < 0.001) for intragroup analyses, considering the same surface treatment and different assessment times.

Regarding ALP expression levels, no statistically significant difference was observed for all implant surfaces after 7 days of healing (MAC = 1.00 ± 0.05, DAE = 0.95 ± 0.05, and NHA = 0.94 ± 0.05) (*p* > 0.106). In contrast, after 30 days, ALP expression showed statistically significant higher levels for NHA (NHA 30D = 1.18 ± 0.05)-coated implants compared to DAE (DAE 30D = 0.70 ± 0.05) and MAC (MAC 30D = 0.27 ± 0.05) implants, both also statistically significant different (*p* < 0.001). Concerning intragroup comparisons, ALP expression levels significantly increased from 7 to 30 days of healing for NAH implants (*p* < 0.001), though its expression levels significantly decrease in a temporal perspective for MAC and DAE implants (*p* < 0.001) (Figure 5).

#### 3.2.3. Osteopontin (OPN)

**Figure 6:**
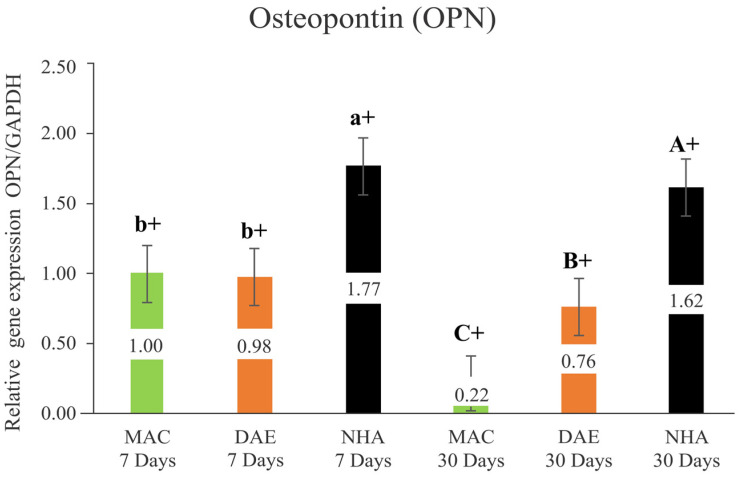
Relative expression of Osteopontin/GAPDH (Opn/GAPDH) in all groups (mean + 95% confidence interval). Lower case letters represent the analysis of 7 days, and capital letters the analysis of 30 days. Different letters indicate a statistically significant difference between groups. The + sign indicates a statistically significant difference (MAC: *p* < 0.001; DAE: *p* < 0.001; NHA: *p* = 0.001) for intragroup analyses, considering the same surface treatment and different assessment times.

The relative expression of OPN after 7 days and at 30 days of healing demonstrated significantly higher levels for the NHA group (NHA 7D = 1.77 ± 0.05/NHA 30D = 1.62 ± 0.05) compared to the DAE (DAE 7D = 0.98 ± 0.05/DAE 30D = 0.76 ± 0.05) and MAC groups (MAC 7D = 1.00 ± 0.05/MAC 30D = 0.22 ± 0.05) (*p* < 0.001). While the OPN expression levels around DAE and MAC implants showed no significant difference at 7 days (*p* = 0.586), DAE presented statistically significant higher OPN levels relative to MAC at 30 days (*p* < 0.001). All implant surface treatments demonstrated a significant reduction in the OPN expression levels from 7 to 30 days of healing (*p* < 0.001) (Figure 6).

#### 3.2.4. Osteoprotegerin (OPG)

**Figure 7:**
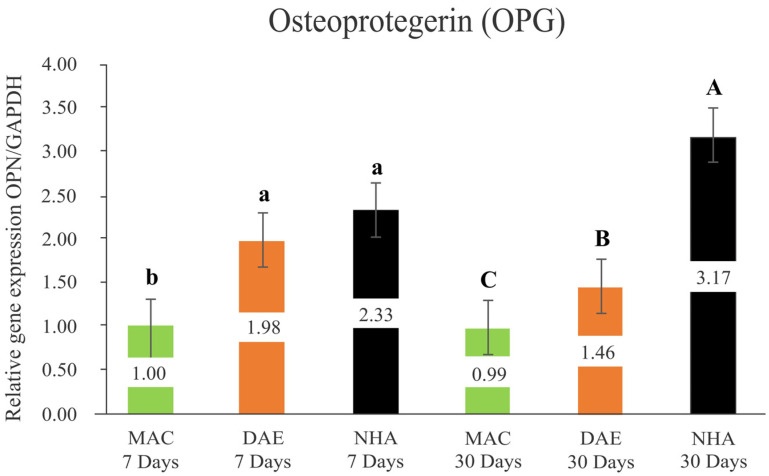
Relative expression of osteoprotegerin/GAPDH (OPG/GAPDH) in all groups (mean + 95% Confidence Interval). Lower case letters represent the analysis of 7 days, and capital letters the analysis of 30 days. Different letters indicate a statistically significant difference between groups. There were not statistically significant differences (*p* ≥ 0.05) for intragroup analyses, considering the same surface treatment and different assessment times.

After 7 days, the relative expression of OPG demonstrated no significant difference between NHA (NHA 7D = 2.33 ± 0.33) and DAE (DAE 7D = 1.98 ± 0.33) surface treatments (*p* = 0.135); however, both presented significantly higher levels of OPG relative to MAC implants (MAC 7D = 1.00 ± 0.33) (*p* < 0.001). After 30 days of healing, OPG expression showed statistically significant higher levels for NHA (NHA 30D = 3.17 ± 0.33)-coated implants compared to DAE (DAE 30D = 1.47 ± 0.33) and MAC (MAC 30D = 0.99 ± 0.33) implants, both also statistically significant different (*p* < 0.047). While OPG expression levels maintained constant from 7 to 30 days for MAC implants (*p* = 0.135), OPG levels significantly decreased for DAE implants and increased for NHA-coated implants (*p* < 0.035) (Figure 7).

#### 3.2.5. Receptor Activator of the Nuclear Factor Kappa Ligand (RANKL)

**Figure 8:**
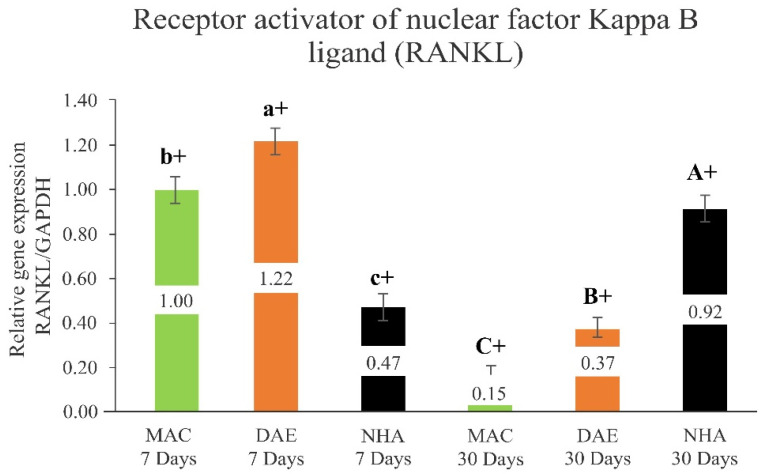
Relative expression of the activating receptor of nuclear factor Kappa ligand (RANKL)/GAPDH (RANKL/GAPDH) in all groups (mean + 95% confidence interval). Lower case letters represent the analysis of 7 days, and capital letters the analysis of 30 days. Different letters indicate a statistically significant difference between groups. The + sign indicates a statistically significant difference (MAC: *p* < 0.001; DAE: *p* < 0.001; NHA: *p* < 0.001) for intragroup analyses, considering the same surface treatment and different assessment times.

The relative expression of RANKL indicated significantly higher expression levels for DAE implants (DAE 7D = 1.22 ± 0.06), followed by MAC (MAC 7D = 1.00 ± 0.08) and NHA-coated (NHA 7D 0.47 ± 0.08) implants, both statistically significant different, after 7 days of healing (*p* < 0.001). In contrast, NHA RANKL expression levels (NHA 30D = 0.92 ± 0.08) demonstrated the highest values, followed by DAE (DAE 30D = 0.37 ± 0.08) and MAC (MAC 30D = 0.16 ± 0.08) implants after 30 days in vivo, all statistically significant different (*p* < 0.001). In a temporal perspective, MAC and DAE RANKL expression levels significantly decreased from 7 to 30 days, while NHA RANKL expression significantly increased from 7 to 30 days (*p* < 0.001) (Figure 8).

#### 3.2.6. RANKL/OPG Ratio

**Figure 9:**
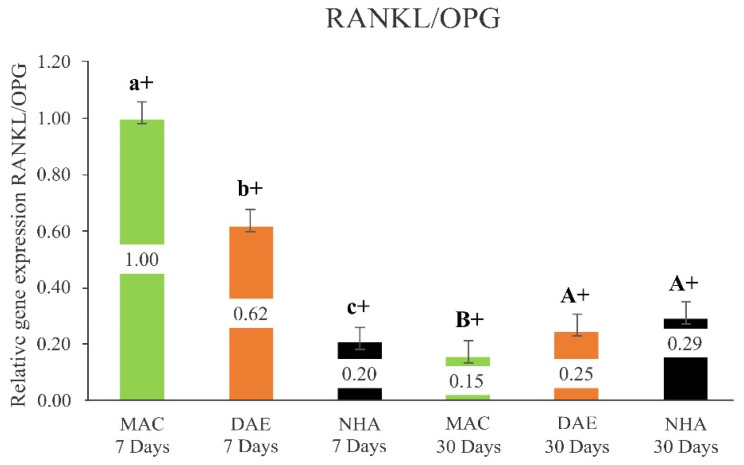
Relative expression of (RANKL/OPG) ratio/GAPDH in all groups (mean + 95% confidence interval). Lower case letters represent the analysis of 7 days, and capital letters the analysis of 30 Days. Different letters indicate a statistically significant difference between groups. The + sign indicates a statistically significant difference (MAC: *p* < 0.001; DAE: *p* < 0.001; NHA: *p* = 0.008) for intragroup analyses, considering the same surface treatment and different assessment times.

When comparing the relative expression of RANKL/OPG at 7 days, the MAC group had the highest levels (MAC 7D = 1.00 ± 0.06), followed by the DAE (DAE 7D = 0.62 ± 0.06) and NHA (NHA 7D = 0.20 ± 0.06) groups (*p* < 0.001). In contrast, at 30 days, the NHA and DAE groups showed significantly higher levels of RANKL/OPG expression (NHA 30D = 0.29 ± 0.06/DAE 30D = 0.25 ± 0.06, *p* = 0.357) relative to MAC implants (MAC 30D = 0.15 ± 0.06) (*p* < 0.021). A significant reduction in the RANKL/OPG expression levels was observed for all implant surface treatments from 7 to 30 days of healing periods (*p* < 0.001) (Figure 9).

#### 3.2.7. Osteocalcin (OCN)

**Figure 10:**
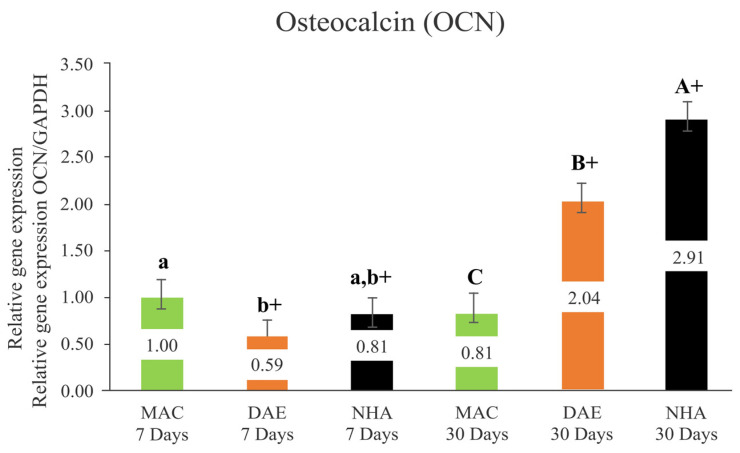
Relative expression of Osteocalcin/GAPDH (OCN/GAPDH) in all groups (mean + 95% confidence interval). Lower case letters represent the analysis of 7 days, and capital letters the analysis of 30 days. Different letters indicate a statistically significant difference between groups. The + sign indicates a statistically significant difference (MAC: *p* = 0.140; DAE: *p* < 0.001; NHA: *p* < 0.001) for intragroup analyses, considering the same surface treatment and different assessment times.

After 7 days of healing, MAC (MAC 7D = 1.00 ± 0.19) implants presented significantly lower levels of OCN expression relative to DAE (DAE 7D = 0.59 ± 0.19) (*p* = 0.004); however no significant difference was observed between MAC and NHA (NHA 7D = 0.81 ± 0.19, *p* = 0.145) implants, as well as between DAE and NHA implants (*p* = 0.102). At 30 days, gene expression of OCN demonstrated higher levels for the NHA group (NHA 30D = 2.91 ± 0.19) when compared to DAE (DAE 30D = 2.04 ± 0.19) and MAC (MAC 30D = 0.81 ± 0.19) implants, all showed a statistically significant difference (*p* < 0.001). While OCN expression levels for MAC implants remained constant from 7 to 30 days of healing (*p* = 0.140), the OCN expression of the DAE and NHA implants significantly increased from 7 to 30 days (*p* < 0.001) (Figure 10).

## 4. Discussion

The current study evaluated the effect of smoke exposure on the relative expression of genes associated with the bone metabolism around implants with a nanohydroxyapatite particle coating (NHA), dual acid etching (DAE), and machined (MAC) in a rat model after 7 and 30 days in vivo. The obtained results indicated significantly higher expression levels of Runx2, OPN and OPG for the NHA-coated implants relative to DAE and MAC implants after 7 days of healing. Similarly, NHA-coated implants showed significantly higher levels of Runx2, ALP, OPN, OPG, OC, RANKL and RANKL/OPG relative to DAE and MAC implants. These data indicated a greater osteogenic activity and high osteoclastic activity around implants with an NHA-coated surface when compared to implants with DAE and MAC surfaces. Thus, the postulated null hypothesis that implant surface treatment would not influence gene expression levels in the presence of smoke exposure was rejected.

Determining plasma cotinine levels has been considered an accurate method to report the efficacy of smoking induction [51]. In humans, cotinine levels of approximately 7 ng/mL have been considered a cut-off concentration to distinguish nonsmokers from smokers [52]. A previous study that sought to develop a rat model of cigarette smoke exposure has shown that cotinine levels of approximately 14 ng/mL differentiate smokers from nonsmokers [53]. In the current study, plasma cotinine levels progressively increased from baseline to 30, 37 and 60 days of the experiment to up to 73.37 ng/mL, indicating a successful effect of the proposed protocol for smoke inhalation, where the deleterious effects of cigarette smoke were already present at the time of implant placement (30 days of smoke induction: 51.20 ng/mL cotinine concentration).

Several studies have demonstrated a strong negative correlation between osteoblastic differentiation and cigarette smoke exposure, or other forms of interaction with cigarette smoke chemicals, which can be represented by a reduction in the relative expression of specific genes, including Runx2, ALP, OPN, OPG and OC [33,54,55]. The mentioned set of genes were significantly overexpressed around NHA implants relative to DAE and MAC implants, especially after 30 days of healing, showing the potential positive effect of a nanostructured hydroxyapatite coating on hastening osseointegration, especially in compromised healing scenarios such as those faced by heavy smokers [43].

Runx2 has been considered a biomarker in the early stages of bone formation, where a high expression level of mRNA is reported after 7 and 14 days of healing, with a significant decrease after 21 days [56]. Similarly, the values obtained by qRT-PCR in the present study indicated a high expression of Runx2 in the early stages of osseointegration, statistically higher for the NHA surface relative to DAE and MAC surfaces. Despite the fact that Runx2 expression significantly decreased in the late phase of osseointegration (30 days) for DAE and MAC surfaces, similar to the above-mentioned study results, Runx2 expression around the NHA surfaces significantly increased in the late stage of healing. The rationale behind this result may lie in the function of Runx2 in osteoblastic differentiation, which is essential for bone formation and skeletal morphogenesis, accelerating bone repair [57].

ALP also plays an important role in osteoblast differentiation and activity, important for osteoid formation and matrix mineralization [30,31,32]. The quantitative analysis of ALP expression of the current study demonstrated similar levels for all implant surfaces in the early healing; however, in the later timepoint, MAC and DAE surfaces showed a decrease in ALP levels, while NHA-coated surfaces presented an increase in the ALP levels. Previous studies have also demonstrated a tendency to a gradual decrease in the ALP expression levels after the first week of implant placement [56]; though NHA complex surfaces have demonstrated not only higher concentration of ALP relative to non-complex implant surfaces but also increased expression in the late phase of osseointegration [32].

OPN is a non-collagenous extracellular matrix protein involved in cell adhesion, migration and regulation of mineral deposition [58,59]. OPN expression around the NHA surface was statistically superior to other surfaces either at 7 or 30 days, where the decrease in the OPN expression levels in the later healing timepoint was meaningfully higher for MAC and DAE surfaces. These findings are in line with those found by De Oliveira and Nanci, 2004, which have shown significantly higher gene expression for nanotextured surfaces when compared to non-complex surface treatments, especially in the earlier stage of bone formation.

OPG is a decoy receptor for RANKL that prevents osteoclastogenesis by blocking the interactions of RANKL with RANK [33,37,38,57]. OPG levels have previously been shown to be significantly reduced in the presence of smoke exposure; in contrast, RANKL levels tend to increase after smoke exposure, which contributes to RANKL–RANK binding [33,60]. In the present study, the NHA-coated surface showed higher OPG expression levels both after 7 and 30 days of healing when compared to the other surfaces, corroborating with the findings of Cyprus et al., 2018, where microstructured surfaces presented higher OPG levels relative to machined surfaces. The increased expression of OPG in complex implant surfaces, especially in the nanostructured surfaces, has been of particular interest to inhibit osteoclast differentiation since OPG binds to RANKL [33,57]. NHA-coated surfaces showed the lowest RANKL expression levels at 7 days, though they presented the highest levels at 30 days. Similarly, quantitative analysis of RANKL/OPG expression showed the lowest level for the NHA group relative to the other groups in the early phase of healing, though NHA group presented the highest expression levels at the late healing phase. Previous studies have also shown a tendency towards the increased expression of OPG and RANKL in the later stages of bone healing, possibly indicating an increase in the bone remodeling process [56].

OCN is a strong indicator of osteoblast differentiation, temporal expression and maturation of extracellular matrix proteins, such as fibronectin or type I collagen, which are essential for bone mineralization [61,62,63,64]. Regarding the quantitative analysis of gene expression, OCN levels significantly increased between 7 days and 30 days for DAE and NHA surfaces, both significantly higher than MAC implants at the late healing timepoint. This result is in agreement with findings in the literature, where higher expression levels of OCN have been reported around nanostructure calcium phosphate-coated surfaces when compared to conventional surfaces [33,63].

This paper is the first to investigate this nanohydroxyapatite surface in animals exposed to cigarette smoke, evaluating the gene expression in bone healing. The literature has shown that this study model presents a deficiency in bone repair, due to the inhibition of proliferation, chemotaxis and cell adhesion, in addition to impaired vascularization and tissue oxygenation [10,15]. The results found in a recent study indicated that this NHA surface showed, in healthy and systemically compromised animals, an increased gene expression, especially in the early stages of osseointegration [45]. Similar results with upregulation for Runx2, ALP and OC were also found in healthy humans after the placement of nanotextured mini-implants when compared to machined implants [65]. In this way, the results found in the present study are in accordance with those previously reported for subjects without smoke exposure.

Altogether, it is important to emphasize the results obtained in the present study concerning the relative superiority of bone formation gene expression around NHA-coated surfaces in comparison to DAE and MAC surfaces, respectively, in an animal model with a potential compromised bone repair due to cigarette smoke exposure. The analysis of longer healing periods is warranted to further investigate the relative gene expression around complex implant surfaces in the osseointegration course; as well as the analysis of the proteins of the osteogenic cascade that are signaled by gene expression, verifying the extent of genetic activation transcribed into protein. Highly translational preclinical models should also be performed to validate the current results in large animals exposed to cigarette smoke. In addition, the clinical extrapolation of the current results is discouraged, and randomized controlled trials are required to confirm the advantages of the NHA surface in humans.

## 5. Conclusions

The expression levels of the set of genes analyzed in rats exposed to smoke inhalation in the current study indicated a greater osteogenic activity and high osteoclastic activity around implants with a dual acid-etched nanohydroxyapatite-coated surface when compared to implants with merely dual acid-etched and machined surfaces. Such results indicate a higher potential for bone repair of the nanostructured hydroxyapatite-coated surfaces relative to the others, and a possible correlation between the expression of the genes examined and the exposure to smoking.

## Data Availability

The data presented in this study are available on request from the corresponding author. The data are not publicly available due to ethical reasons.
